# Enhancing Stem Cell-Based Therapeutic Potential by Combining Various Bioengineering Technologies

**DOI:** 10.3389/fcell.2022.901661

**Published:** 2022-07-05

**Authors:** In-Sun Hong

**Affiliations:** ^1^ Department of Health Sciences and Technology, GAIHST, Gachon University, Seongnam, South Korea; ^2^ Department of Molecular Medicine, School of Medicine, Gachon University, Seongnam, South Korea

**Keywords:** stem cells, biomaterials, scaffolds, microenvironment, therapeutic effects

## Abstract

Stem cell-based therapeutics have gained tremendous attention in recent years due to their wide range of applications in various degenerative diseases, injuries, and other health-related conditions. Therapeutically effective bone marrow stem cells, cord blood- or adipose tissue-derived mesenchymal stem cells (MSCs), embryonic stem cells (ESCs), and more recently, induced pluripotent stem cells (iPSCs) have been widely reported in many preclinical and clinical studies with some promising results. However, these stem cell-only transplantation strategies are hindered by the harsh microenvironment, limited cell viability, and poor retention of transplanted cells at the sites of injury. In fact, a number of studies have reported that less than 5% of the transplanted cells are retained at the site of injury on the first day after transplantation, suggesting extremely low (<1%) viability of transplanted cells. In this context, 3D porous or fibrous national polymers (collagen, fibrin, hyaluronic acid, and chitosan)-based scaffold with appropriate mechanical features and biocompatibility can be used to overcome various limitations of stem cell-only transplantation by supporting their adhesion, survival, proliferation, and differentiation as well as providing elegant 3-dimensional (3D) tissue microenvironment. Therefore, stem cell-based tissue engineering using natural or synthetic biomimetics provides novel clinical and therapeutic opportunities for a number of degenerative diseases or tissue injury. Here, we summarized recent studies involving various types of stem cell-based tissue-engineering strategies for different degenerative diseases. We also reviewed recent studies for preclinical and clinical use of stem cell-based scaffolds and various optimization strategies.

## Introduction

Previous studies demonstrates that stem cell transplantation ameliorates tissue damage and robustly regenerates diseased or injured organs via various repair mechanisms (Moeinabadi-Bidgoli et al., 2021). Currently, various types of stem cells, such as induced pluripotent stem cells (iPSCs) (Netsrithong and Wattanapanitch, 2021), embryonic stem cells (ESCs) (Lan et al., 2020), and mesenchymal stem cells (MSCs) (Hernandez et al., 2020) have been shown to enhance tissue repair/regeneration by modulating immune reactions and/or direct differentiation into target cells. Although stem cell-based therapeutic approaches have emerged as a promising alternative for the treatment of various degenerative diseases, their full-scale clinical application is limited by the relatively low cell viability at the sites of injury of the transplanted cells (Zhang S. et al., 2020) and their poor multilineage differentiation into target tissues (Park JS. et al., 2021). For example, neural stem cell transplantation is widely used to treat ischemic brain injury or neurodegenerative disease (Zhao L. et al., 2021); however, their differentiation into fully functional cells of neural lineage and stable engraftment, followed by reconnection with host neural cells at the injured site are still obstacles to overcome (Kwon et al., 2018; Katoh et al., 2019).

To overcome their current limitations, biomaterial-based tissue engineering using many different types of biocompatible cell-seeded scaffolds has been tried to enhance the cell viability, growth potential, and multilineage differentiation of transplanted stem cells (Xu et al., 2020; Belludi et al., 2021; Gogele et al., 2022; Zheng et al., 2022). A number of naturally derived and synthetic biomaterials have been currently developed to restore the function and structure of damaged tissues (Park S. R. et al., 2021; Mazzoni et al., 2021). Indeed, various biodegradable protein-based natural polymers, including collagen, fibrin, gelatin, hyaluronic acid, and poly (lactic-co-glycolic) acid have been widely used to construct scaffolds for the regeneration of multiple tissues such as bone, cartilage, ligament, neural tissues, skin, skeletal muscle, and blood vessels (Rice et al., 2013; Wang et al., 2020; Bonferoni et al., 2021).

The biomaterial scaffold-based approaches are primarily designed to incorporate living therapeutic cells within a porous 3D scaffold and signaling molecules or growth factors by providing a tissue architecture for cell infiltration and proliferation to promote the repair and regeneration of damaged tissue (Salerno and Netti, 2021). For example, the combination of several biodegradable 3D scaffolds and transplanted stem cells is an attractive therapeutic strategy for the regenerations of injured tissues by facilitating cell survival and retention (Abbott et al., 2012; Dash et al., 2018; Nagano et al., 2021). Therefore, in this article, we will critically discuss recent advances and limitations of various stem cell-based tissue-engineering therapies and their preclinical or clinical application in multiple types of disease for tissue regeneration.

## Bioengineering Techniques Accelerate Stem Cell-Based Therapeutic Effects

**FIGURE 1:**
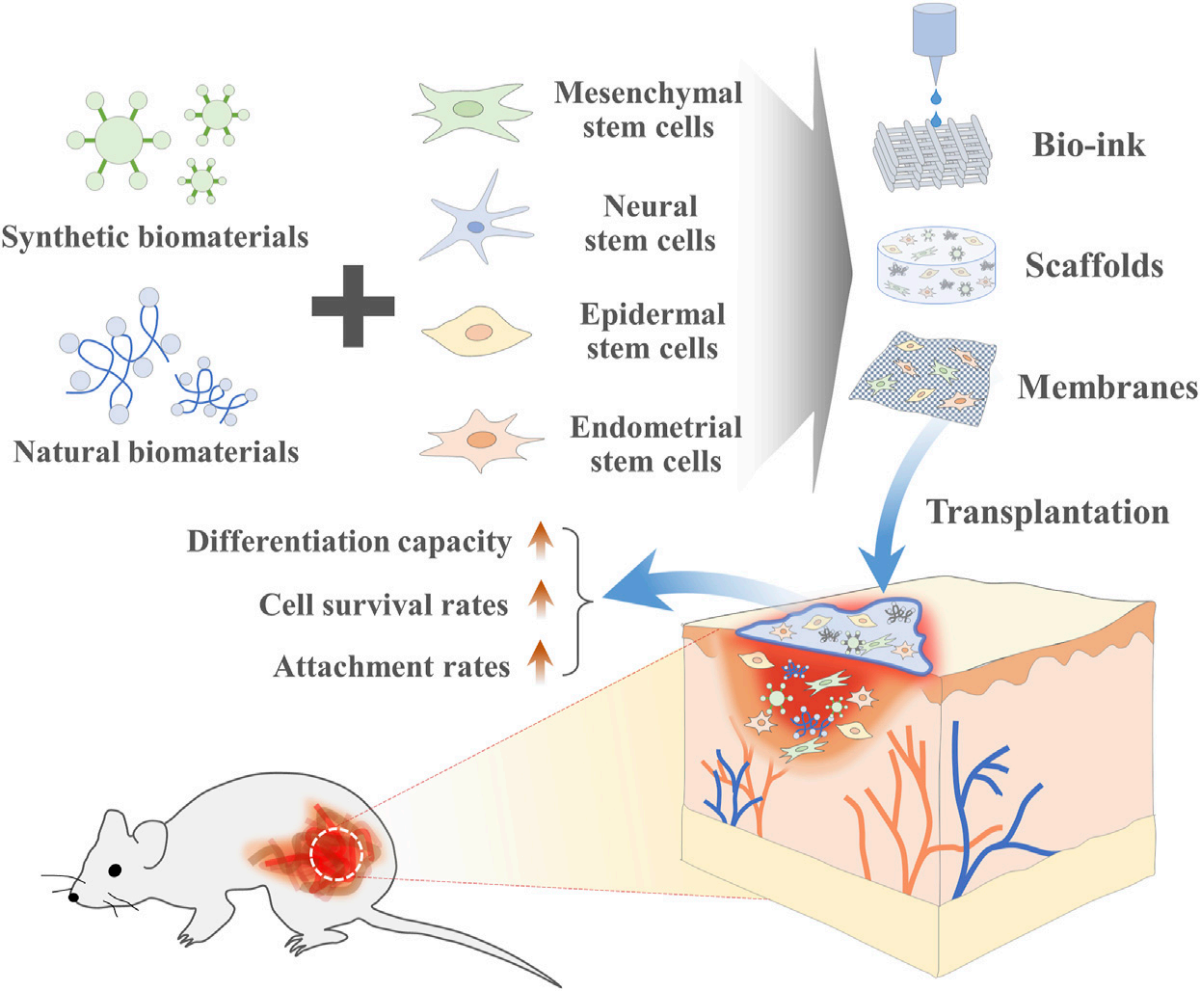
The combination of stem cells and biomaterial-based tissue engineering can promote regenerative capacity of stem cells. Various synthetic or natural biomaterials-based 3-dimensional (3D) scaffolds can be used to overcome various current limitations of stem cells-based therapies by supporting their adhesion, survival, proliferation, and differentiation as well as providing elegant 3D tissue microenvironment at the sites of injury.

Due to their relatively high self-renewal capacity, the ability to differentiate into multiple cellular lineages, and immunomodulatory properties, various types of stem cells, especially MSCs have been extensively investigated over the past decade as a promising cell resource in a number of preclinical and clinical studies to treat or ameliorate various incurable diseases, such as cirrhosis, diabetes, inflammatory disease, liver failure, and neurodegenerative disorders (Chen J. et al., 2021; Chen X. et al., 2021). Despite substantial progress in stem cell therapies, many challenges remain to be overcome prior to their therapeutic application. Regardless of the source, one of the major bottlenecks of their clinical application is the large-scale *in vitro* expansion prior to transplantation to obtain adequate numbers of stem cells, normally millions to hundreds of millions of stem cells required per patient (Wei et al., 2014; Garcia-Bernal et al., 2021). Therefore, 3D porous or fibrous biomaterials-based scaffold can be used to overcome these limitations by supporting their adhesion, survival, proliferation, and differentiation as well as providing elegant 3-dimensional (3D) tissue microenvironment (Summarized in Figure 1).

Scaffolds based on synthetic or natural biomaterials provide various chemical cues and mechanical supports for the transplanted stem cells at specific sites of injured tissues. Typically, Gilbert et al. (2010) loaded muscle stem cells to the tunable polyethylene glycol (PEG) hydrogel platform to properly regenerate damaged skeletal muscle by increasing their survival rates and self-renewability in the sites of injury. In addition, synthetic peptide-acrylate surfaces support self-renewal of human ESCs with similar morphology and phenotypic marker expression compared to cells cultured on Matrigel (Melkoumian et al., 2010). Kim et al. (2010) also developed synthetic peptide substrates that recognize cell surface glycosaminoglycan for long-term *in vitro* expansion of human pluripotent stem cells. In addition, the maintenance of stem cells in their undifferentiated state during *in vitro* cell expansion, efficient control of stem cell differentiation into target tissues both pre- and post-administration, and adequate protection of the stem cells during and after transplantation to patients are challenges (Neofytou et al., 2015; Liu et al., 2020). Various natural or synthetic biomaterial-based tissue engineering techniques may offer novel solutions to overcome these obstacles.

A specific microenvironment that provides signaling factors and elements plays a significant role in regulating cell survival and differentiation (Zhang et al., 2019). In this context, to effectively mimic the *in vivo* microenvironment, combining various biomaterials with stem cells is a powerful tool for controlling stem cell viability and fate by facilitating cell-matrix/cell-cell interactions (Gelmi and Schutt, 2021). In contrast to traditional 2D cell culture conditions, the 3D culture platform using biomaterial-based scaffolds provides a more *in vivo* physiologic architecture for implanted stem cells by supporting physical interactions with extracellular matrix (ECM) components and releasing signaling molecules (Li et al., 2017). Therefore, chemical, electrical, mechanical, surface topography, and structural characteristics need to be comprehensively evaluated when manufacturing a biomaterials-based scaffold (Martino et al., 2012). In particular, efficient *ex vivo* expansion and differentiation of implanted stem cells into target tissues on synthetic or natural microcarriers largely depends on their potential to regulate cell shape (spreading or round) and cell organization (aggregate size) (Sart et al., 2013). Microcarrier techniques are often used in bioreactors to promote stem cell expansion *in vitro* (Bardy et al., 2013; Li et al., 2014). For example, Bardy et al. (2013) achieved approximately 7-fold increased expansion of human iPSCs with significantly elevated differentiation into neural progenitor cells using a matrigel-based microcarrier (MC) culture system.

In addition, the highly hydrated and microporous hydrogels, a class of biomaterial scaffolds, provide ideal 3D microenvironments to stably encapsulate stem cells together with signaling molecules, peptides, or growth factors (Aguado et al., 2012; Yan et al., 2012). Hydrogels, made of various natural polymers such as natural polymers like collagen, alginates, gelatin, and hyaluronic acid, serve as immunological barriers to protect the implanted stem cells from host immune attack while maintaining permeability to therapeutic molecules or growth factors (Nafea et al., 2011). In addition, Gaffey et al (2015) reported that endothelial progenitor cells, which are seeded into hyaluronic acid (HA) shear-thinning hydrogel and then transplanted into the ischemic rat hearts, resulted in enhanced engraft efficiency and reduced myocardial fibrosis compared with stem cells treated with normal saline. Injectable hydrogels based on peptide amphiphiles also significantly enhanced the transplantation of muscle stem cells in injured muscles by ensuring growth factor retention and thus increasing proliferation of encapsulated cells (Sleep et al., 2017).

## The Correlation Between the Physical Properties of Biomaterials and Their Target Tissue

Because different tissues or cells have their own chemical compositions and physical characteristics, the designed biomaterials used in tissue engineering should have a strong functional and structural affinity for targeted tissue and cell types to properly stimulate tissue regeneration (Abbas et al., 2020; Peressotti et al., 2021). The structural or mechanical properties of the biomaterial surface such as charges, chemical compositions, and hydrophobicity may play key roles in regulating their diverse biological functions (Echeverria Molina et al., 2021; Pearce and O’reilly, 2021). The therapeutic effects are largely dependent on the reciprocal interactions between the transplanted biomaterials with specific mechanical characteristics and 3D microenvironment of targeted tissues (Barthes et al., 2014; Nicolas et al., 2020; Antmen et al., 2021). For example, relatively strong mechanical properties may be required to regenerate hard tissues such as bones or teeth which may be subjected to weight-loading or strain (Mastrogiacomo et al., 2019), whereas porous, soft, and highly viscose biomaterials are needed to restore the functions of the soft tissues such as skin and internal organs (Bonferoni et al., 2021).

In addition, the use of biomaterials also largely depends on the predicted application types (open implantation (attachment) strategies vs internal injection or less invasive treatments) (Kohane and Langer, 2008; Qi et al., 2015; Raucci et al., 2020). Generally, biomaterial-based tissue mimetics are fully fabricated outside the body (*in vitro*) and then should be transplanted surgically. Therefore, for such applications, the whole tissue mimetics need to have relatively low viscosity with certain level of mechanical strength that can be implanted. Conversely, biomaterials fabricated for internal injection have relatively high viscosity and soft mechanical strength with more cohesive and gel-like characteristics. Currently, a number of studies have investigated the effects of structural (mechanical) and chemical characteristics of biomaterials on various cellular functions such as self-renewability, migratory capacity, and metabolic activity (Ma et al., 2019; Zhao X. et al., 2021; Peressotti et al., 2021).

### Regulation of Stem Cell Microenvironment With Biomaterial Scaffolds

Tissue resident stem cells are located in particular 3D microenvironment, defined as niches to interact with neighboring cells, matrix components, bound or secreted biomolecules, and growth factors, which are essential for their sufficient therapeutic effects after administration (He et al., 2014). Interestingly, various synthetic or natural biomaterial-based scaffolds can provide a longer and more efficient cellular microenvironment for administrated stem cells retention and survival rates in injured tissues and subsequently enhance their therapeutic potential after administration. Indeed, insulin like growth factor-1 (IGF-I) conjugated fibrin micro-beads significantly increased bulk stability and muscle regeneration capacity of human smooth muscle cells (Vardar et al., 2019).

Kim et al. (2021) also observed that adipose-derived stem cells, which were self-assembled with the cartilage-dECM-decorated nanofibrils, enhanced their survival rates and differentiation capacity and subsequently enhanced therapeutic efficiency in osteochondral defect mice by mimicking structural and biochemical cartilage-specific microenvironment. In addition, the combination of chitosan-coated silicone tube and neurosphere cells induced from human adipose-derived MSCs increased their myelinated axons density and myelin thickness and thus exhibited substantial improvements in nerve regeneration after 6 weeks of surgery (Hsueh et al., 2014).

Similarly, hydrogel comprised of thiol-functionalized hyaluronic acid and hyperbranched poly (ethylene glycol) diacrylate (HB-PEGDA) polymer significantly enhances cell growth, survival, and paracrine activity of adipose-derived MSCs and subsequently accelerated wound closure and reduced the scar formation in burn skin wound model (Dong et al., 2020). Supramolecular nanofibers containing arginine-glycine-aspartate (RGD) peptides can bind strongly to extracellular vesicles (EVs) derived from MSCs. This EV-RGD hydrogels significantly attenuated histopathological damage, decreased tubular injury and in turn increased therapeutic efficacy in renal repair in early phases of acute kidney injury (Zhang C. et al., 2020).

Various types of growth factors (cytokines) can increase stem cell proliferation, migratory capacity, and differentiation potential towards a specific lineage (Meng et al., 2008). In this context, Ferguson et al. conjugated epidermal growth factor (EGF) and basic fibroblast growth factor (bFGF) with dextrin to support growth and differentiation of mouse neural stem cells via controllable growth factor release (Ferguson et al., 2018). Similarly, Zhao et al. synthesized a chitosan hydrogel scaffolds by conjugating C domain peptide of IGF-1 onto chitosan and co-transplanted with human placenta-derived MSCs into a murine hindlimb ischemia model.

Transplanted scaffolds substantially reduced fibrosis and collagen deposition, along with increased angiogenesis by protecting H_2_O_2_-induced apoptosis and decreasing inflammation (Zhao J et al., 2019). In addition, nitric oxide (NO), a short-lived and highly reactive free radical, is an essential intra- and extracellular messenger with critical signaling roles in regulating diverse cellular functions associated with tissue regeneration (Midgley et al., 2020). However, the short biological half-life of NO (5–10 s) and diffusion distance severely limited its therapeutic application in stem cell-based regenerative medicine (Hughes et al., 2009). Thus, Nie et al. (2017) developed a controllable chitosan NO-releasing hydrogel (CS-NO) to promote direct endothelial differentiation of mouse ESCs by activating PI3K/Akt signaling pathway without adding growth factors. Similarly, Zhang et al. developed NO-releasing chitosan hydrogel to increase proangiogenic capacities of human placenta-derived MSCs and thus enhance their therapeutic efficiency in hindlimb ischemia model (Zhang K. et al., 2020).

## Regeneration and Repair of Skin Wounds

The skin is the largest organ in the human body and is made up of three basic layers: dermis, epidermis, and hypodermis or subcutaneous tissue. It supports and protects the underlying skeletal muscles, bones, ligaments, internal organs, connective tissues (Bhardwaj et al., 2017). It contains many specialized cells and complex multi-layered structures with different mechanical behaviors (Biggs et al., 2020). Skin provides the first line of defense against any pathogenic infection as an immune-protective organ (Di Meglio et al., 2011). Thus, skin is the most vulnerable to infection and requires rapid wound healing. Application of stem cell technologies in wound healing and skin regeneration has increased considerably in recent decade (Kucharzewski et al., 2019; Nourian Dehkordi et al., 2019). Several distinct stem cell populations have been identified and characterized in the epidermis with distinct locations and functions (Rognoni and Watt, 2018), and strong skin repair and regenerative abilities (Yang et al., 2020). Stimulating the local stem cell populations and promoting specific microenvironment (termed stem cell niche) that enhances their self-renewal capacity and differentiation potential may accelerate the regeneration and repair of injured skin (Wong et al., 2012; Fuchs and Blau, 2020).

Scaffolds based on synthetic or natural biomaterials provide various chemical cues and mechanical supports for the transplanted stem cells at specific sites of injured skin (Riha et al., 2021; Weng et al., 2021). For example, although G-protein coupled receptor 6-positive (LGR6^+^) epithelial stem cell populations were isolated from the follicular bulge and substantial wound-healing effects were achieved when transplanted into full-thickness cutaneous wounds (Lough et al., 2014), it resulted in poor therapeutic efficacy with limited epithelialization, hair growth, and angiogenesis. Therefore, to address these limitations, Denver et al. developed LGR6^+^ epithelial stem cell-seeded scaffold to provide deliverable, immediate and viable soft tissue mimetics that significantly improved the rates of healing, epithelialization, angiogenesis or vasculogenesis, and hair growth in full-thickness wounds (Lough et al., 2016).

In addition to LGR6^+^ epidermal stem cells, MSCs derived from various tissues including bone marrow, umbilical cord blood, and adipose tissue (Laverdet et al., 2014) are also extensively used to regenerate injured skin. Indeed, MSC-transplanted wounds exhibited significantly accelerated cutaneous healing with increased re-epithelialization, vascularization, and differentiation into epithelial cells and keratinocytes (Wu et al., 2007; Sasaki et al., 2008). Bone marrow (BM)-derived MSC-seeded collagen-chitosan scaffolds having mechanical properties similar to that of the soft tissues significantly improved the degree of collagen deposition and wound healing rates (Abolgheit et al., 2021). Formigli et al. (2015) also demonstrated that BM-MSCs seeded on synthetic polysiloxane polymer-based scaffold largely ameliorated cutaneous wounds, enhanced reepithelization and collagen deposition, and promoted vascularization and hair growth at the implantation sites by releasing various paracrine factors and/or recruiting endogenous progenitor cells.

Adipose tissue derived (AD)-MSCs or secretome significantly enhanced the healing process in early phase by increasing the migration and proliferation of dermal fibroblasts and inhibiting the inflammatory response (Ma et al., 2021). These intracutaneously injected AD-MSCs at the dermal and subcutaneous layers can survive *in vivo* for up to 1 year in immunodeficient mice and differentiate into adipocytes to provide architectural support and microenvironment for re-epithelialization and subsequent dermal repair (Koellensperger et al., 2014). Furthermore, AD-MSCs implanted into the curcumin-loaded collagen scaffold markedly accelerated ulcer healing process (Mardani et al., 2020). Similarly, Lotfi et al. (2019) demonstrated that the combination of AD-MSCs and keratinocytes on gelatin/chitosan/β-glycerol phosphate nanoscaffold can be used in cutaneous wound healing by reducing wound size as well as increasing angiogenesis of the dermis and dermal thickness.

Artificial dermal substitutes, such as atelocollagen matrix, seeded with AD-MSCs were accurately incorporated into the regenerating capillaries, dermis, and epidermis at the site of wound healing in diabetic db/db mice (Nambu et al., 2011). In addition, silk fibroin scaffolds cellularized with human Wharton’s jelly derived-MSCs enhanced myofibroblast proliferation and re-epithelialization as well as reduced scar tissue formation and inflammatory infiltration in models of skin wound healing (Millan-Rivero et al., 2019). Human umbilical cord blood (UCB)-derived MSCs seeded on collagen-fibrin double-layered scaffolds exhibiting a highly porous and interconnected structure also significantly accelerated wound healing in mouse models of full-thickness skin wounds (Nan et al., 2015).

In addition, epidermolysis bullosa (EB) is a severe skin disease associated with skin fragility, which is caused by different genetic mutations of some genes involved in regulating adhesion of basal epidermal cells to the underlying basement membrane (Jackson et al., 2017; Alharthi et al., 2021). Therefore, gene therapeutic strategies linked to EB can be applied to induce tissue regeneration of EB skin lesions or prevent EB-indued wound healing abnormalities (Marinkovich and Tang, 2019; Gurevich et al., 2022). In this context, eenetically modified skin-derived stem cells have great potential as successful cell-based gene delivery systems locally or systemically. Indeed, several clinical trial observed that local implantation of transgenic epidermal stem cells can regenerate a functional epidermal layer, leading to permanent closure of a large chronic wound of EB skin lesions (Mavilio et al., 2006; De Rosa et al., 2014; Bauer et al., 2017). Similarly, Hirsch et al. (2017) also regeneration of the entire human epidermal layer by transplantation of transgenic stem cells into 7-year-old child suffering from life-threatening form of EB. Although these genetically modified skin stem cells could achieve a certain degree of therapeutic effects, the development of an effective gene transfer technology that can make a sufficient therapeutic effects remains a challenge to be overcome. In this context, development of novel biomaterials is being tried to effectively deliver therapeutic genes to target cells that constantly are involved in the regeneration of the dynamic epidermal tissue. For example, several positively charged polycationic molecules, such as polybrene, which has been widely used in many clinical studies, have applied to increase *in vivo* retroviral gene delivery efficiency. Recently, Barbier et al. (2022) developed a system for the efficient therapeutic gene transduction to keratinocytes and dermal fibroblasts *in vivo* using EF-c, that forms amyloid-like peptide nanofibrils. Similarly, Dakiw Piaceski et al. (2018) also combined therapeutic gene (COL7A1 gene, encoding type VII collagen) and tissue-engineered skin substitute for treating dystrophic EB.

## Bone Regeneration and Repair

Previous studies have shown the biocompatibility, degradability, mechanical performance, and porosity of tissue-engineered 3D scaffolds for accelerated bone regeneration in various bone defect models (Zhang H.-X. et al., 2016; Lai et al., 2018; Liu et al., 2019). In addition, the use of biomaterial-based scaffolds for the local delivery of stem cells into bone defect sites decreases the potential risk of ectopic bone formation. The 3D structure of the scaffolds mimics the natural bone microenvironment and thus facilitates cell adhesion, growth, differentiation (Baker and Chen, 2012; Ahmad et al., 2018). Bone tissue bioengineering based on natural or synthetic biomaterial 3D scaffolds requires a promising stem cell source to successfully accelerate bone regeneration.

Various types of stem cells with osteogenic differentiation capacity *in vivo*, such as MSCs, ESCs, and iPSCs, have been used in 3D scaffold-based bone tissue engineering (Mizuno, 2009; Grassel and Lorenz, 2014). In particular, various tissue-derived MSCs are attractive candidates due to their high self-renewal and osteogenic differentiation potential (Rao and Stegemann, 2013). Indeed, MSCs play a critical role in bone regeneration and repair by differentiating into bone-forming osteoblasts to produce bone matrix (Florencio-Silva et al., 2015) and chondrocytes that subsequently undergo mineralization and resorption upon implantation (Knight and Hankenson, 2013) and thus appear to be a better cell source for 3D scaffold-based bone bioengineering than iPSCs and ESCs (Battafarano et al., 2021). Indeed, Cidonio et al. (2020) developed nanocomposite-based 3D scaffolds seeded with human BM-MSCs and umbilical vein endothelial cells (HUVECs) to closely recapitulate the architecture and function of bone tissue and ultimately promote skeletal differentiation. This BM-MSCs-laden nanoclay-based 3D printed scaffolds were found to significantly improve vascular ingrowth and bone tissue formation *in vivo* compared with acellular scaffolds (Cidonio et al., 2020). Redondo et al. (2018) also performed a pilot clinical trial using a cross-linked serum scaffold (BioMax) seeded with autologous BM-MSCs for treatment of maxillary cysts in nine patients. No inflammatory response or other serious adverse effects were found, and the density of cyst interior remarkably increased after the transplantation of this scaffold (Redondo et al., 2018).

Various bioengineering techniques have been used to integrate stem cells on the scaffold. For instance, Sartika et al. (2020) directly seeded AD-MSCs onto previously fabricated 3D silk fibroin (SF) scaffold with relatively high cell viability and enhanced proliferation. Another approach involved incorporating stem cells simultaneously with nanoclay-based bioinks during the fabrication of scaffold by Cidonio et al. (2020). Similarly, Qiao et al. (2021) fabricated a tri-layered stratified scaffold in which BM-MSCs were simultaneously integrated with gelatin methacrylamide (GelMA) hydrogel using a UV-light assisted, stepwise infiltration and crosslinking method.

In addition, a significant improvement in bone regeneration in the presence of stem cells has recently been demonstrated with various types of composites, ceramics, metals, and polymeric materials (Cidonio et al., 2020; Lozano et al., 2020; Volkov et al., 2020). Indeed, it has recently been shown that Zn^2+−^releasing *β*-tricalcium phosphate/poly (l-lactic acid) (TCP/PLLA) ceramics with periosteum-derived progenitor cells represent ideal scaffolds for bone regeneration and repair given their immunoregulatory capacity and unique osteoinductive activity (Huang et al., 2021). Similarly, Marcacci et al. (2007) transplanted a porous hydroxyapatite (HA) ceramic scaffold seeded with patient-derived BM-MSCs into four patients with large bone diaphysis defects, resulting in effective and long-term bone regeneration. Peng et al. (2011) also fabricated biphasic calcium phosphate (BCP) ceramic scaffolds using a 3D gel-lamination technique to seed autologous BM-MSCs *in vitro*. They implanted the BCP scaffold into canine models with bone defects, resulting in significantly greater osteointegration and new bone formation than in the absence of BMSC seeding scaffold.

Regarding the use of polymers associated with muscle stem cells, Peter et al. fabricated a polyglycolic acid mesh seeded with multipotent muscle stem cells, which were isolated from adult skeletal muscle, and found significantly enhanced therapeutic effects after transplantation into a calvarial defect rat model (Taub et al., 2009). Harada et al. (2014) also fabricated a copolymer poly (D, L-lactic-co-glycolic acid) (PLGA) scaffold seeded with cartilage-forming chondrocytes pre-differentiated *in vitro* from rat BM-MSCs and evaluated bone formation efficiency. Volkov et al. (2020) reported therapeutic effects of biocompatible and biodegradable polymer scaffolds, poly (3-hydroxybutyrate) with hydroxyapatite filled with alginate hydrogel, containing BM-MSCs in a rat model of parietal bone defect. Although the *in vitro* and *in vivo* toxic effects on human cells are currently under investigation (Sherry et al., 1989; Zhang XF. et al., 2016; Escarcega-Gonzalez et al., 2018), Zhang et al. (2015) demonstrated that silver nanoparticles (AgNps) encapsulated with collagen accelerated bone fracture healing by facilitating growth and osteogenic differentiation of MSCs and facilitating chemo-attraction of MSCs and fibroblasts into the site of injury.

## Spinal Cord or Peripheral Nerve Injury Regeneration

Neural stem cell administration without a supporting ECM results in poor cell viability, low differentiation potential into neural lineage cells, and low transplantation rates at sites of spinal cord injury (Cao et al., 2001; Zhu et al., 2018). To overcome the limitations of neural stem cell therapy in spinal cord or peripheral nerve injury regeneration, tissue bioengineering-based regeneration and repair strategies received increased attention during the past decades. Based on this bioengineering approach, various natural and synthetic biomaterial-based scaffolds combined with different types of stem cells have been developed for direct transplantation into injured regions to regenerate and restore spinal cord or peripheral nerve function.

Chitosan, a chitin-deacetylated non-toxic product, been used as the tube biomaterial to facilitate axonal regeneration and to promote neural stem differentiation into neurons after spinal cord injury (Rao et al., 2018; Liu H. et al., 2021). For example, Li et al. (2009) developed chitosan carriers combined with well-known neurotrophic factor neurotrophin-3 (NT-3), which enhanced the viability and differentiation of neural stem cells into neurons. The chitosan carriers exhibited good biocompatibility with the neural stem cells and significantly promoted the differentiation of neural stem cells into neurons including cholinergic and GABAergic neurons. Similarly, Nomura et al. (2008) implanted chitosan channels seeded with neural stem cells between the cord stumps after complete spinal cord transection. They reported tissue bridge formation and increased astrocytic and oligodendrocytic differentiation in the chitosan channels in the spinal cord at 14 weeks after transplantation (Nomura et al., 2008).

Collagen is the most abundant protein constituting approximately 30–40% of the total protein in human body and a substantial portion of the ECM (Leon-Lopez et al., 2019). It is found in the proliferative regions during neurogenesis and suppresses proliferation and glial cell differentiation while increasing neuronal differentiation of neural progenitor cells (Ali et al., 1998). Kourgiantaki et al. (2020) demonstrated that porous collagen-based scaffolds (PCS) seeded with embryonic neural stem cells can be used to effectively transplant and protect implanted cells at sites of spinal cord injury for enhanced neuronal differentiation, leading to significantly improved therapeutic effects in mouse model. Zou et al. (2020) also fabricated a collagen sponge scaffold with human spinal cord-derived neural stem cells, and the transplantation of this scaffold effectively increased long-term cell survival and neural differentiation and improved the microenvironment at the site of spinal cord injury by decreasing inflammatory response and glial scar formation. A multi-channel collagen scaffold with axially aligned luminal conduits, which is loaded with neural stem cells, promotes neural stem cell activity and enhances cell proliferation without changing cell differentiation potential, thereby enhancing spinal cord repair following injury (Liu S. et al., 2021).

In addition, hyaluronic acid is a non-immunogenic non-sulfated anionic natural polysaccharide with highly conserved structure and biocompatibility that is present throughout the body including cartilage, soft connective tissues, neural tissues, skin, synovial fluids, and umbilical cords (Deepa et al., 2006; Burdick and Prestwich, 2011). It is a major component of the extracellular matrix and plays an important role in regulating angiogenesis, cell differentiation, cell signaling, matrix organizations, and tissue regeneration (Allison and Grande-Allen, 2006; Li et al., 2012), and therefore represents a key biomaterial in regenerative medicine (Shahi et al., 2020).

Three-dimensional (3D) biodegradable porous scaffolds with hyaluronic acid are central elements in bioengineering-based tissue regeneration (Zhai et al., 2020). Zarei-Kheirabadi et al. (2020) transplanted embryonic neural stem cells with commercially available hyaluronic acid-based hydrogel into the spinal cord injury rat to induce differentiation of these cells into astrocytes, neurons, and oligodendrocytes leading to increased neuronal myelination. Similarly, Arulmoli et al. (2016) found that scaffolds containing a combination of fibrin with interpenetrating networks of hyaluronic acid and laminin significantly promoted the growth and differentiation of human neural stem/progenitor cells while attenuating cell-mediated degradation. The scaffolds also enhanced vessel formation and complexity of human cord blood-derived endothelial cells when co-cultured with neural stem cells (Arulmoli et al., 2016). By incorporating single-walled carbon nanotubes and polypyrrole, Shin et al. (2017) developed porous catechol-functionalized hyaluronic acid hydrogels that significantly improved neuronal differentiation of human neural stem/progenitor cells with increased electrophysiological functionality. Calcium channel expression, intracellular, and calcium influx depolarization of loaded neural stem cells were markedly increased in 3D electroconductive hydrogels.

## Vascularized Tissue Regeneration and Repair

Cardiovascular diseases are one of the leading causes of morbidity and mortality in developing countries and are rapidly increasing globally. As extensively reviewed and discussed by Zaragoza et al. (2011), both environmental and genetic factors play an important role in the pathogenesis of cardiovascular disease and its related disorders, which are complex and multifactorial and extremely difficult to treat.

Various biomaterial-based tissue engineering techniques have been suggested as alternative therapeutic strategies to overcome the limitations of current *treatment in* cardiovascular disease by directly transplanting vascularized cardiac tissue mimetics, which are fabricated according to the specific tissue microenvironment at the sites of injury (Nugent and Edelman, 2003). Selecting the appropriate cell source reflecting tissue-specific features is also important for the development of functional cardiac or vascular tissue mimetics, to ensure structural stability and accurate incorporation into the damaged sites (Vunjak-Novakovic et al., 2010).

Other major challenges associated with the application of vascular cells in cardiovascular diseases are the poor quality and quantity of proliferating cells, which deteriorates with the donor’s age and also depends on the selected cell type and isolation method (Janzen et al., 2006; Mimeault and Batra, 2009). In this context, various types of stem/progenitor cells are being used as sources of endothelial cells, smooth muscle cells, and vascular cells to facilitate vascular regeneration and incorporation into the engineered vasculature (Tsifaki et al., 2018; Vila Cuenca et al., 2021). In particular, MSCs from bone marrow or adipose tissues are an attractive source of vascular cells for tissue bioengineering (Pittenger et al., 2019).

Several previous studies reported the increased differentiation potential of AD-MSCs into functional contractile smooth muscle cells (Rodriguez et al., 2006; Heydarkhan-Hagvall et al., 2008; Harris et al., 2011), which were encapsulated with biomaterial-based hydrogels and proliferated on decellularized blood vessel scaffolds, suggesting their capacity for regenerating the vascular structures and functions (Harris et al., 2011). Li et al. (2020) implanted AD-MSCs on atelocollagen scaffolds, induced differentiation into cardiocyte-like cells using 5-azacytidine, and subsequently transplanted into mice with ischemic myocardium. Krawiec et al. (2017) also fabricated biodegradable, elastomeric, porous vascular scaffolds using non-toxic, cytocompatible poly (ester urethane) urea. This tissue-engineered vascular graft was seeded with AD-MSCs and transplanted into the infrarenal abdominal region of rat. Finally, a novel vascular-like tissue *in vivo* was detected by analyzing the ECM collagen and elastin within implanted vascular scaffolds (Krawiec et al., 2017). Decellularized aorta elastin scaffold with biodegradability and cytocompatibility in combination with human AD-MSCs was also developed by Kazemi et al. (2021).

BM-MSCs are recognized as a promising cell source to restore damaged myocardium after myocardial infarction due to their potential for differentiation into functional cardiomyocytes (Khan et al., 2017) and secretion of various paracrine factors (Song et al., 2017). Indeed, seeding of BM-MSCs porous scaffolds using collagen, which is a major constituent of the myocardial ECM, improves myocardial function by increasing trophic factor secretion and regulating immune modulatory function (Rashedi et al., 2017). Gong et al. successfully fabricated a small-diameter biodegradable vessel scaffold using polyglycolic acid (PGA) and BM-MSC-derived endothelial and smooth muscle cells (Gong and Niklason, 2008). Similarly, BM-MSCs seeded on polycaprolactone 3D nanofiber scaffolds induced cardiac lineage differentiation when transplanted into a rat myocardial infarction model in combination with Wnt/β-catenin signaling regulator IWP-2 and the native cardiac protein thymosin β4 (Marks and Kumar, 2015). Liu et al. (2007) embedded BM-MSCs in fibrin hydrogels, which were polymerized on approximately 4-mm diameter cylindrical scaffold to mimic the structure of blood vessels and transplanted into the jugular veins of lambs. The grafted scaffold exhibited a confluent endothelial layer and the embedded BM-MSCs secreted significant amounts of collagen (Liu et al., 2007).

Among human MSCs, umbilical cord (UCB)-derived MSCs are characterized by relatively low immunogenicity and high proliferative potential for *in vitro* expansion prior to clinical application (Li et al., 2015). A clinical trial revealed that UCB-MSCs are safe and effective cell source for patients with myocardial infarction (Gao et al., 2015). UCB-MSCs exhibited their capacity for self-organization into matrigel-mediated cell networks and activated angiogenic myeloid cells (Roura et al., 2012). Ultimately, UCB-MSC-seeded matrigel induced the formation of new functional microvasculature that is connected with the host vascular system in animal models of acute myocardial infarction (Roura et al., 2012). Pushp et al. (2020) seeded UCB-MSC-derived beating cardiomyocytes expressing various cardiac-specific genes on aligned polycaprolactone (PCL) nanofibrous scaffolds. The aligned PCL scaffolds stimulated parallel orientation of the seeded cells with the fibers, thus effectively regulating anisotropic conditions *in vitro* (Pushp et al., 2020).

Previous studies indicated that endothelial progenitor cells (EPCs), characterized by increased differentiation into functional endothelial cells, represent an appropriate source of endothelial regeneration via angiogenesis and re-endothelialization (Walter et al., 2002; Werner et al., 2002; Urbich and Dimmeler, 2004). In this context, Kaushal et al. (2001) isolated EPCs from ovine peripheral blood and seeded them onto decellularized 4 mm blood vessels obtained from porcine iliac arteries. The tissue-engineered small blood vessels showed contractility and nitric oxide-induced vascular contraction/relaxation similar to natural cardiac arteries (Kaushal et al., 2001). Lv et al. (2020) also developed cardiac ECM-chitosan-gelatin seeded with CD34-positive EPCs. It is a highly porous, biodegradable, non-toxic, and biocompatible 3D scaffold, which supported reendothelialization of seeded EPCs (Lv et al., 2020).

## Tracheal Tissue Regeneration via Stem Cell-Based Tissue Engineering

The native trachea is a hollow tube composed of multiple cartilage rings interspersed with well-vascularized fibrous connective tissue (Udelsman et al., 2018). It is lined by pseudostratified ciliated columnar epithelium and plays an essential role in respiration and indirectly swallowing (Udelsman et al., 2018). Tracheal epithelium also plays a significant role in host defense by eliminating various environmental insults, such as pathogenic bacteria, dust particles, and mold (Nomoto et al., 2006). Narrowing or constriction of the trachea can lead to life-threatening respiratory emergencies because airway stenosis can prevent sufficient air supply to the lungs. Therefore, innovative interventions and treatments for tracheal regeneration have been developed by surgeons using biomaterial and tissue bioengineering technologies.

Current therapeutic approaches for tracheal repair and regeneration include cell-free 3D tissue mimetics (Omori et al., 2005; Tatekawa et al., 2010), autologous tissues (Fabre et al., 2013; Ch’ng et al., 2014), and decellularized autologous tissues, which retain the native organ ultrastructure and are re-cellularized by the recipient-derived cells (Jungebluth et al., 2012a; Elliott et al., 2012; Berg et al., 2014; Haykal et al., 2014). Despite the wide variety of therapeutic strategies, these approaches are limited by low availability, insufficient mechanical strength, and morphological changes during or after transplantation, which can cause a narrowing or blockage of airway (Ch’ng et al., 2014).

In addition, allogeneic grafts increase the risk of disease transmission (microbiological contamination) and immune reactions against donor MHC, suggesting the need for immunosuppressive therapies to appropriately block the immune response against donor antigens in xenogeneic tissues. Due to these limitations, implantation of various fully differentiated allogeneic airway cells into the bioengineered tracheal grafts may be difficult clinically. In this context, stem cells have received substantial attention as an alternative cell source, because not only do they support tissue regeneration but also exhibit significant immunosuppressive function to prevent undesired immune response.

Currently, decellularized trachea is one of the optimal scaffolds, due to its increased pro-angiogenic activities, excellent biocompatibility, and original tubular structure (Jungebluth et al., 2012b). For example, Yao et al. (2021) developed decellularized tracheal scaffolds with vascularized BM-MSC sheet showing strong angiogenic potential. Similarly, Elliott et al. (2017) seeded autologous BM-MSCs onto decellularized tissue-engineered tracheal scaffold and transplanted into a patient with a long-segment congenital tracheal stenosis after conventional reconstructive techniques failed. Xu et al. (2019) also developed decellularized tracheal alternatives to repair long-segment tracheal defects using the laser micropore technique with BM-MSCs. The tracheal scaffold significantly increased the efficiency of cell attachment in addition to ensuring homogenous cell distribution throughout the scaffold. Additionally, the tissue-engineered trachea promoted chondrogenesis of the BM-MSCs and the formation of homogeneous neocartilage *in vivo* (Xu et al., 2019). Choi et al. (2021) reported that hyaluronic acid-coated polycaprolactone scaffolds resulted in better MSC adhesion rates and greater mucosal regeneration compared with non-coated scaffolds. Dikina et al. (2015) developed high-cell density, scaffold-free BM-MSC-derived cartilaginous rings and tubes, in which TGF β1-delivering gelatin microspheres were incorporated to enhance the chondrogenic differentiation efficiency of MSCs. Xu et al. (2021) developed chondroitin-sulfate-incorporated type-II atelocollagen (COL II/CS) biomimetic tracheas with significant chondrogenic potential using BM-MSCs to facilitate the formation of vascularized fibrous tissues and subsequently mimic the tracheal structure and function. The biomimetic tracheas exhibited successful tracheal reconstruction and showed satisfactory therapeutic outcomes *in vivo* (Xu et al., 2021).

Furthermore, Taniguchi et al. (2018) fabricated a new scaffold-free artificial trachea using bio-3D printing technology with spheroids consisting of various autologous cell types, such as chondrocytes, endothelial cells, and MSCs. This bio-3D-printed artificial tracheas had sufficient strength to transplant and support chondrogenesis and vasculogenesis *in vivo* (Taniguchi et al., 2018). In addition, the two-layered artificial trachea was fabricated with 3D-printed polycaprolactone (PCL) microfibers (outer layer) and electrospun PCL nanofibers (inner layer), and then seeded with iPSCs-derived chondrocytes, iPSCs-derived mesenchymal stem cells, and bronchial epithelial cells (Kim et al., 2020). The artificial trachea enhanced the regeneration of tracheal cartilage and tracheal mucosa in a rabbit model of defective tracheal segment (Kim et al., 2020). However, tissue-engineered tracheal transplantation is mainly limited by tracheal collapse associated with chondromalacia of transplantation cartilage (Kobayashi et al., 2010). Chondromalacia of tissue-engineered trachea might be mostly attributed to poor chondrogenic differentiation of seeded stem cells, insufficient cell retention or survival, and inadequate vascularization of transplanted trachea. To address the chondromalacia of transplantation cartilage, Yan et al. designed a new cell sheet scaffold using poly (trimethylene carbonate) (PTMC) and poly (lactic-co-glycolic acid) (PLGA) to fabricate a porous membrane structure for implanting BM-MSCs. Four weeks after transplantation of tissue-engineered trachea, the number of differentiated chondrocytes, the amount of cartilage matrix, and vascularization were significantly increased (Yan et al., 2017).

## Reproductive Tract Tissue Regeneration

Naturally thin endometrial thickness or mechanical damage to the endometrial basal layer during artificial abortion may lead to implantation failure and subsequent female infertility (Revel, 2012). Although several pharmacological strategies, including steroid hormones (Sharma et al., 2018) and chemokines/growth factors (Yi et al., 2018) have been used to regenerate the uterine tissue, these therapeutic strategies failed to sufficiently increase endometrial receptivity and subsequent pregnancy outcomes. Therefore, the development of new alternative therapeutic approaches to increase uterine receptivity is imperative. In this context, substantial efforts have been devoted to restore thin or injured endometrium via transplantation of various types of stem cells (Nagori et al., 2011; Singh et al., 2014; Santamaria et al., 2016; Zhao N et al., 2019). Despite positive preliminary results, regenerating the damaged endometrium by administrating stem cells is a huge challenge, due to the lack of an appropriate niche specific for the transplanted stem cells within the tissues, leading to poor therapeutic outcomes.

One of the most promising strategies includes development of artificial microenvironments for endometrium via tissue engineering technologies using endometrial scaffolds. In this context, Xin et al. (2019) developed a collagen scaffold loaded with human UCB-MSCs, which promoted endometrial regeneration and restored fertility by promoting collagen remodeling, intrinsic endometrial cell growth, and the expressions of estrogen and progesterone receptors. Arezoo et al. (2021) developed decellularized uterine scaffolds loaded with menstrual blood stem cells, which differentiated into various endometrial cell types. Miyazaki et al. developed a rat decellularized uterine matrix via aortic perfusion with detergents. The decellularized endometrial tissue was then reseeded with adult and neonatal endometrial cells and MSCs (Miyazaki and Maruyama, 2014). The recellularized uterine matrix was transplanted into a partially excised uterine tissue, leading to successful endometrial regeneration and subsequent pregnancy outcomes almost comparable to normal endometrium (Miyazaki and Maruyama, 2014). Similarly, Campo et al. (2017) decellularized and subsequently recellularized porcine uterine extracellular matrix disks using a human stem cells. Tiemann et al. (2020) also decellularised sheep uterus while maintaining a high integrity of the extracellular components and recellularised with heterogeneous sheep fetal bone marrow stem cells.

In addition, Abbas et al. (2020) generated porous collagen scaffolds loaded with multi-cellular endometrial organoids containing both endometrial stromal and epithelial cells. These seeded cells developed a luminal epithelial layer on the surface of the porous scaffold (Abbas et al., 2020). Recently, Park et al. developed a multiple endometrial stem cell-laden artificial endometrium using two biodegradable natural polymers such as hyaluronic acid and collagen to recapitulate the multicellular and complex structure of endometrial tissue (Park S. R. et al., 2021). Severe tissue injuries were successfully resolved via implantation of stem cell-laden artificial endometrium into a mouse model of endometrial damage (Park S. R. et al., 2021).

The ovary is a highly complex and unique female reproductive organ producing an optimal number of mature oocytes with developmental competence via folliculogenesis and ovulation (Ding et al., 2017). Currently, approximately 1% of women develop premature ovarian failure (POF), also called primary ovarian insufficiency (POI) characterized by hypergonadotropic amenorrhea and subsequent depletion (dysfunction) of ovarian follicles before the age of 40 years (Welt, 2008). Various types of stem cells have been used in an attempt to restore ovarian function in animal models of premature ovarian insufficiency (Sun et al., 2013). For example, endometrial stem cells improve ovarian function, such as oocyte production and serum anti-Müllerian hormone in a rodent model of chemotherapy-induced POF (Reig et al., 2019). However, the stem cell-based therapeutic approaches are restricted by insufficient survival and attachment of administered stem cells within the target sites. Administered therapeutic cells diffused passively to other organs or surrounding tissues (Suuronen et al., 2006; Sun et al., 2013).

Therefore, to overcome these limitations, Su et al. (2016) developed a porous and biodegradable collagen-based matrix for migration and function of transplanted adipose-derived stem cells. Transplanted stem cells with collagen improved ovarian function impaired by chemotherapy by increasing their survival and attachment rates within the ovary, and thereby alleviated premature ovarian insufficiency in rodent models. In addition, the ECM also acts as a reservoir for various bioactive molecules, such as cytokines and growth factors, which stimulate follicular development, growth, and maturation (Sadr et al., 2018; Tomaszewski et al., 2021). Thus, decellularized ovarian ECM-based scaffold provides an optimal and native 3D microenvironment to enhance the therapeutic effects of administered stem cells. Indeed, Pennarossa et al. (2021) produced a decellularized ovarian bioscaffold, which mimics the microarchitecture and biological signals in natural ovarian tissue, and the differentiated mature ovarian cells derived from female germline stem cells were repopulated on decellularized bioscaffolds. Similarly, Hassanpour et al. (2018) developed a human decellularized ovarian scaffold seeded with Wharton jelly-derived MSCs. This ovary-specific scaffold served as a native niche to stem cells and successfully improved ovarian function by increasing serum estradiol and progesterone levels in an ovariectomized animal model (Hassanpour et al., 2018). Similarly, Yang et al. (2019) reported that collagen scaffold loaded with human UCB-MSCs improved ovarian function in mice with premature ovarian failure by enhancing the secretion of estrogen and anti-Mullerian hormone (AMH).

## Stem Cell-Based Engineering Combined With Nanotechnology

Stem cell therapies have extraordinary potential for promoting regenerative medicine. However, their clinical application is largely restricted by the lack of effective methods for long-term *in vivo* monitoring of transplanted cells and insufficient therapeutic effects *in vivo* (Dong et al., 2021). The integration of stem cell biology with nanotechnology will potentially improve differentiation capacity, survival rates, and attachment, which in turn will significantly enhance the therapeutic outcomes of stem cell therapies in various incurable diseases (Wang et al., 2009).

Effective isolation of undifferentiated cell populations is considered crucial for successful stem cell-based therapeutics. Lui et al. (2013) developed a new method to effectively isolate stem/progenitor cells from the brain using CD133 antibodies conjugated to magnetic nanoparticles (Ab-MNPs), which develop into neurospheres and differentiate into various cell types. Similarly, Jing et al. (2007) selectively isolated CD34^+^ blood progenitor cells from circulation by CD34 antibody-conjugated magnetic nanoparticle with a purity of 60–96% using the continuous quadrupole magnetic flow sorter (QMS).

Highly advanced nanostructure-based scaffolds or hydrogels for optimal regulation of stem cell behavior have recently received considerable attention in regenerative medicine (Abdal Dayem et al., 2018; Zhang et al., 2021). Nanomaterial-based therapeutic approaches have been designed using various biodegradable and biocompatible nano-carriers or nano-fibers such as self-assembling peptides (Marchini et al., 2020), carbon nanotubes (Lalwani et al., 2017; Naskar et al., 2017), collagen nanoparticles (Bagher et al., 2016), graphene-oxide nanofibers (Zhou et al., 2019), polycaprolactone (PCL) (Lee et al., 2013), tricalcium silicate (C3S) (Jung et al., 2020), and tricalcium phosphate (TCP) (Nuschke et al., 2016) to promote stem cell differentiation and therapeutic outcomes. For example, Chuang et al. (2021) reported that titanium dioxide (TiO_2_) nanoparticles combined with polybutadiene substrates have a synergistic effect in stimulating proliferation and differentiation of dental pulp stem cells into bone or tooth cells.

Li et al. (2016) also developed a series of bovine serum albumin (BSA)-coated gold (Au) nanospheres with diameters of 40, 70 and 110 nm. The gold nanospheres showed good cytocompatibility without influencing the proliferation of human MSCs and promoted osteogenic differentiation (Li et al., 2016). Similarly, Ko et al. (2015) also designed gold nanoparticles with varying size (15, 30, 50, 75 and 100 nm) and similar surface chemistry and investigated their role in cytotoxicity, proliferation, and osteogenic differentiation of AD-MSCs.

Various carbon nanotube-based scaffolds have been designed to promote stem cell attachment, expansion, and differentiation into specific lineages (Nayak et al., 2010; Baik et al., 2011; Lee et al., 2014). For example, Nayak et al. (2010) developed thin films of pegylated multiwalled carbon nanotubes as porous scaffolds for human MSCs. The functionalized carbon nanotube-based scaffolds did not show cytotoxicity and significantly accelerated osteogenic differentiation of human MSCs (Nayak et al., 2010). Lee et al. (2014) also fabricated carbon nanotube-collagen hydrogels to mimic 3D microenvironment and promote neural differentiation of BM-MSCs and secretion of neurotrophic factors. In addition, carbon nanotubes have recently been utilized in other biomedical applications, such as bioimaging, biosensing, and drug delivery involving various bioactive agents (Harrison and Atala, 2007; Saito et al., 2009). These nanoparticles have been used to identify and track transplanted cells *in vivo*, and to improve the efficiency of stem cell-based therapy. Well-known nanoparticles for long-term *in vivo* tracking of transplanted stem cells include quantum dots (Kundrotas et al., 2019), magnetite nanoparticles (Guldris et al., 2017), and gold nanorods (Yu et al., 2021). Carboxylated quantum dots localize mainly to the perinuclear region of human BM-MSCs without adversely affecting their viability, and thus can be used as nonspecific and effective dyes for staining of BM-MSCs (Kundrotas et al., 2019).

Gold nanoparticles have also been widely used in computed tomography (CT) imaging and tracking of transplanted human MSCs because of their excellent biocompatibility and strong photoelectric absorption coefficient (Yu et al., 2021). Interestingly, stem cell-conjugated nanoparticles, which are loaded with therapeutic agents, can also be used as an effective photodynamic platform against tumor cells. For instance, the two-cycle photoactivations of BM-MSCs loaded with positively charged poly-methyl methacrylate core-shell fluorescent nanoparticles (FNPs) significantly induced apoptosis of osteosarcoma cells in both 2D and 3D conditions (Lenna et al., 2020). Recently, nanoparticles were used as non-viral gene delivery systems for stem cells.

Genetic modifications of stem cells have been extensively used to improve their paracrine secretion of certain growth factors, survival rates, and lineage-specific differentiation *in vivo*, which subsequently enhanced the therapeutic effects of administrated stem cells (Hodgkinson et al., 2010). Traditional viral vector systems have been widely utilized to stimulate delivery and stable expression of specific therapeutic genes with high efficiency in host cells. However, their clinical application is still restricted by possible oncogenic potential, strong immunogenicity, and limited gene-loading capacity (Lehrman, 1999; Walther and Stein, 2000). For example, Green et al. (2008) developed positively charged (∼10 mV) and small (∼200 nm), biodegradable polymeric nanoparticles to enhance nonviral gene delivery efficiency up to 4-fold higher than that of commercially available transfection agents in human ESCs. Xu et al. (2018) also developed iron oxide nanoparticles (IONPs) as an optimal gene delivery platform, which significantly improves the self-renewal capacity and the multi-lineage differentiation potential of human mesenchymal stem cells.

## 3D Bioprinting With Stem Cell Technology

3D bioprinting is a tissue-engineering technology that allows precise layer by layer deposition of biocompatible materials, live cells, and supporting components (referred to as bioinks) via a computer-controlled process to mimic complex 3D structural architecture and subsequently generate functional tissues or organs (Groll et al., 2016; Moroni et al., 2018). The development of tissue-specific bioinks provides an optimized 3D microenvironment with precise positioning of living cells layer by layer and thus mimics biological and physical properties of native tissues, which in turn facilitates repair of tissue defects and restoration of tissue structure and function (Murphy and Atala, 2014; Mandrycky et al., 2016; Lee et al., 2018). In this context, 3D bioprinting techniques combined with various biomaterials and growth factors overcome the low therapeutic efficacy of stem cell-based therapies for various human diseases by supporting their specific niche and micro-architecture, which influences the cell fates and survival rate (Skeldon et al., 2018).

Currently, multipotent MSCs are one of the most popular stem cell types used in 3D bioprinting, probably due to the relative ease to culture *in vitro* and high self-renewability, multipotency, and high safety (low immunogenicity and tumorigenicity) compared with other types of stem cells (Skeldon et al., 2018). Zhu et al. (2022) developed 3D-bioprinted bone-like tissue scaffolds using BM-MSCs and multifunctional nanocomposite bioink consisting of alginate dialdehyde-gelatin and mesoporous bioactive glass nanoparticles. Similarly, Costantini et al. (2016) fabricated hydrogel cartilage scaffolds loaded with 3D-bioprinted BM-MSCs using a bioink consisting of chondroitin sulfate amino ethyl methacrylate, gelatin methacrylamide, and hyaluronic acid methacrylate. They reported significantly enhanced viability and chondrogenic differentiation of loaded BM-MSCs within the fabricated hydrogel cartilage scaffolds (Costantini et al., 2016). Restan Perez et al. (2021) also designed neural tissues loaded with patient-derived AD-MSCs using fibrin-based bioink and microfluidic RX1 3D bioprinter, and analyzed the expression of various neural markers, dopamine release, and electrophysiological activity. Daly et al. (2016) fabricated hypertrophic cartilage templates loaded with BM-MSCs with vascularization and mineralization using gamma-irradiated alginate bioink incorporating Arg-Gly-Asp adhesion peptides (). Furthermore, the mechanical properties of this soft cartilage tissue can be reinforced with a network of 3D-printed polycaprolactone fibers. Du et al. (2015) bioprinted BM-MSC-laden methacrylamide gelatin scaffolds carrying collagen-binding domain (CBD)-BMP2-collagen microfibers at the micrometer scale. The CBD-BMP2-collagen microfibers effectively induced differentiation of BM-MSCs into osteogenic lineage within 14 days (Du et al., 2015).

Current 3D bioprinting technology facilitates printing of iPSC-derived differentiated cells or undifferentiated iPSCs mixed with bioinks derived from various biomaterial (Maiullari et al., 2018; Yeung et al., 2019). Importantly, 3D bioprinting of autologous iPSC-derived cells or tissues may not cause immune rejection or infection with organ transplants. Therefore, Kawai et al. (2021) recently developed scaffold-free tubular heart tissues using 3D bioprinting techniques with iPSCs-derived cardiomyocytes, human umbilical vein endothelial cells, and human fibroblasts. The beating of these 3D-bioprinted heart tissues was observed in mice with clear striations of the myocardium and vascularization after 1 month post-transplantation (Kawai et al., 2021). Zhang et al. used 3D-bioprinted endothelial cells within microfibrous hydrogel scaffolds to form a layer of confluent endothelium (Zhang Y. S. et al., 2016). The endothelialized 3D scaffolds were then seeded with human iPSC-derived cardiomyocytes to establish the endothelialized-myocardium organ-on-a-chip for cardiovascular toxicity evaluation (Zhang Y. S. et al., 2016). Ma et al. (2016) 3D bioprinted hydrogel-based microscale hepatic constructs loaded with human iPSCs-derived hepatic progenitor cells, AD-MSCs, and human umbilical vein endothelial cells. This 3D-bioprinted hepatic construct improved the expression of various liver-specific genes, metabolic product secretion, and cytochrome P450 expression (Ma et al., 2016).

In addition, biomimetic tissue-like constructs for neural engineering were developed using 3D bioprinting technologies for neurodegenerative diseases (Thomas and Willerth, 2017). In this context, Sharma et al. (2020) fabricated small spherical neural particles using microfluidics-based RX1 bioprinter with fibrin-based bioink and iPSC-derived neural progenitor cells. These 3D-bioprinted neural tissues expressed various neuronal markers (TUJ1, FOXA2, NURR1, TH, and PAX6) and glial markers (GFAP and O4) (Sharma et al., 2020). Jury et al. (2022) also developed 3D-bioprinted neural structures with hyaluronan and poly (ethylene glycol)-based hydrogels. The neuroepithelial stem cells within hydrogels undergo spontaneous differentiation into neural cells with significantly enhanced proliferation and viability. Huang et al. (2017) developed a graphene–polyurethane composite hydrogel as a potential bioink for enhanced survival and differentiation of neural stem cells within 3D-bioprinted neural tissue structures. The graphene-based hydrogel significantly increased the oxygen metabolism and the neural lineage differentiation of loaded neural stem cells (Huang et al., 2017). Liu et al. 3D-bioprinted neural stem cell-laden scaffolds, which maintain high cell viability (about 95%) and facilitate neural lineage differentiation for optimized neural networks (Liu X. et al., 2021). The 3D-bioprinted neural tissue structure significantly enhanced axon regeneration and reduced glial scar deposition *in vivo* (Liu X. et al., 2021).

## Clinical Trials of Stem Cell-Based Bioengineering Therapeutics

Currently, various stem cell-based tissue engineering products provides novel clinical therapeutic opportunities for a number of degenerative diseases or tissue injury. For example, collagen sponge scaffolds (Condress^®^, Istituto Gentili, Milano, Italy) seeded with autologous dental pulp stem cells (DPSCs) were transplanted into the chronic periodontitis patients with deep intrabony defect. Various radiographic and clinical parameters were assessed at baseline, 6 and 12 months after surgery. The transplanted sites exhibited significantly more probing depth reduction, bone defect fill, clinical attachment, and periodontal regeneration than control groups (Ferrarotti et al., 2018).

Gjerde et al. (2018) also implanted biphasic calcium phosphate scaffolds loaded with bone marrow-derived MSCs into the resorbed alveolar ridge in the patients with severe mandibular ridge resorption. After 4–6 months of healing, new bone formation was assessed radiographically and clinically. The loaded MSCs could expand on the scaffolds and successfully induce significant formation of new bone, without adverse events.

In addition, Ding et al. (2018) observed that umbilical cord-derived MSCs on a collagen-based scaffold can restore the functions of primordial follicles through FOXO1 and FOXO3a signaling pathways *in vitro*. Transplantation of this collagen scaffold loaded with MSCs to the dormant ovaries of premature ovarian failure (POF) patients reactivated various ovarian functions, such as estrogen secretion, follicular growth, and ovulation. Successful clinical pregnancy was achieved in patients with POF after implantation of this collagen scaffold loaded with MSCs.

Recently, Meamar et al. seeded human placenta-derived MSCs onto the electrospun gelatin nanofibrous scaffolds (GNS) and cultured with platelet-rich plasma (PRP) for 7 days. This scaffold then implanted to the patients with diabetic foot ulcers (DFUs) and then various clinical parameters were assessed after 12-week. Indeed, wound healing, new capillary formation, and pain-free walking distance were significantly increased by the implantation of placenta-derived MSCs seeded GNS (Meamar et al., 2021).

## Discussion

Currently, a number of biomaterials and multiple types of stem cells are available to mimic the structure, architecture, and function of specific tissues. Several studies continue to increase our understanding of transplanted tissue mimetics and their development *in vivo* to identify the stem cell type for clinical application. Further studies are needed to provide comprehensive insight into the growth and integration of stem cell-loaded tissue mimetics with host tissues. Indeed, various synthetic or natural biomaterial-based scaffolds can provide a longer and more efficient cellular microenvironment for administrated stem cells retention and survival rates in injured tissues and subsequently enhance their therapeutic potential after administration. For example, insulin like growth factor-1 (IGF-I) conjugated fibrin micro-beads significantly increased bulk stability and muscle regeneration capacity of human smooth muscle cells (Vardar et al., 2019).

Although stem cell-based tissue engineering therapeutic strategies have demonstrated successful outcomes in preclinical and clinical trials, several challenges still remain before their clinical application can be envisaged. Indeed, various stem cell-based tissue engineering products provides novel clinical therapeutic opportunities for a number of degenerative diseases or tissue injury. For example, collagen sponge scaffolds seeded with autologous dental pulp stem cells (DPSCs) were transplanted into the chronic periodontitis patients with deep intrabony defect (Ferrarotti et al., 2018). Gjerde et al also implanted biphasic calcium phosphate scaffolds loaded with bone marrow-derived MSCs into the resorbed alveolar ridge in the patients with severe mandibular ridge resorption (Gjerde et al., 2018). However, further studies are needed to address the immunological and related issues, challenges associated with appropriate integration of stem cell-loaded engineered mimetics into host tissues and their high variation in therapeutic efficiency, effects on the surrounding tissues, disease progression, age, or transplantation route. Thus, interdisciplinary research is absolutely necessary before full clinical applications of stem cell-based tissue engineering can be utilized n tissue repair and regeneration.

In addition, the interactions of transplanted stem cells and biomaterial-based scaffolds within the injured tissues are complex and multi-factorial, in addition to the need for an optimal platform to promote the functional restoration following injury. Focused investigations are needed to evaluate the types and combinations of national or synthetic biomaterials required to increase survival rate and multilineage differentiation of transplanted stem cells within 3D porous scaffolds and their interaction with host cells and microenvironment at the site of injury. Interestingly, various synthetic or natural biomaterial-based scaffolds can provide a longer and more efficient cellular microenvironment for administrated stem cells retention and survival rates in injured tissues and subsequently enhance their therapeutic potential after administration. Indeed, IGF-I conjugated fibrin micro-beads significantly increased bulk stability and muscle regeneration capacity of human smooth muscle cells (Vardar et al., 2019). In addition, the combination of chitosan-coated silicone tube and neurosphere cells induced from human adipose-derived MSCs exhibited substantial improvements in nerve regeneration (Hsueh et al., 2014).

Chemical groups, surface structure, physical properties or technique of biomaterial preparation need to be intensively analyzed to regulate the behavior and fate of transplanted stem cells. Because different tissues or cells have their own chemical compositions and physical characteristics, the designed biomaterials used in tissue engineering should have a strong functional and structural affinity for targeted tissue and cell types to properly stimulate tissue regeneration (Abbas et al., 2020; Peressotti et al., 2021). The structural or mechanical properties of the biomaterial surface such as charges, chemical compositions, and hydrophobicity may play key roles in regulating their diverse biological functions (Echeverria Molina et al., 2021; Pearce and O’reilly, 2021).

Further, the characteristics and tissue origin of transplanted stem cells should be determined before their application in the synthesis of biomaterial-based 3D porous scaffolds (Redondo et al., 2017). For example, stem cells derived from tissues with high turnover rates such as bone marrow, intestinal tract, and skin undergo rapid proliferation and regeneration, whereas stem cells isolated from the muscles and liver facilitate tissue regeneration (Redondo et al., 2017). By contrast, stem cells isolated from inactive regenerating tissues such as heart and brain exhibit low proliferation and poor regenerative potential (Redondo et al., 2017).
